# Does the Arcto-Tertiary Biogeographic Hypothesis Explain the Disjunct Distribution of Northern Hemisphere Herbaceous Plants? The Case of *Meehania* (Lamiaceae)

**DOI:** 10.1371/journal.pone.0117171

**Published:** 2015-02-06

**Authors:** Tao Deng, Ze-Long Nie, Bryan T. Drew, Sergei Volis, Changkyun Kim, Chun-Lei Xiang, Jian-Wen Zhang, Yue-Hua Wang, Hang Sun

**Affiliations:** 1 School of Life Science, Yunnan University, Kunming, Yunnan, China; 2 Key Laboratory for Plant Diversity and Biogeography of East Asia, Chinese Academy of Sciences, Kunming, Yunnan, China; 3 University of Chinese Academy of Sciences, Beijing, China; 4 Key Laboratory of Plant Resources Conservation and Utilization, College of Biology and Environmental Sciences, Jishou University, Jishou, Hunan, China; 5 Department of Biology, University of Nebraska at Kearney, Kearney, Nebraska, United States of America; Institute of Botany, CHINA

## Abstract

Despite considerable progress, many details regarding the evolution of the Arcto-Tertiary flora, including the timing, direction, and relative importance of migration routes in the evolution of woody and herbaceous taxa of the Northern Hemisphere, remain poorly understood. *Meehania* (Lamiaceae) comprises seven species and five subspecies of annual or perennial herbs, and is one of the few Lamiaceae genera known to have an exclusively disjunct distribution between eastern Asia and eastern North America. We analyzed the phylogeny and biogeographical history of *Meehania* to explore how the Arcto-Tertiary biogeographic hypothesis and two possible migration routes explain the disjunct distribution of Northern Hemisphere herbaceous plants. Parsimony and Bayesian inference were used for phylogenetic analyses based on five plastid sequences (*rbcL*, *rps16*, *rpl32-trnH*, *psbA-trnH*, and *trnL-F*) and two nuclear (ITS and ETS) gene regions. Divergence times and biogeographic inferences were performed using Bayesian methods as implemented in BEAST and S-DIVA, respectively. Analyses including 11 of the 12 known *Meehania* taxa revealed incongruence between the chloroplast and nuclear trees, particularly in the positions of *Glechoma* and *Meehania cordata*, possibly indicating allopolyploidy with chloroplast capture in the late Miocene. Based on nrDNA, *Meehania* is monophyletic, and the North American species *M. cordata* is sister to a clade containing the eastern Asian species. The divergence time between the North American *M. cordata* and the eastern Asian species occurred about 9.81 Mya according to the Bayesian relaxed clock methods applied to the combined nuclear data. Biogeographic analyses suggest a primary role of the Arcto-Tertiary flora in the study taxa distribution, with a northeast Asian origin of *Meehania*. Our results suggest an Arcto-Tertiary origin of *Meehania*, with its present distribution most probably being a result of vicariance and southward migrations of populations during climatic oscillations in the middle Miocene with subsequent migration into eastern North America via the Bering land bridge in the late Miocene.

## Introduction

The biogeographic history of intercontinental disjunctions between eastern Asia and eastern North America has long fascinated botanists and biogeographers [1–3], but until the inception of molecular phylogenetics and the accompanying advance of complex analytical approaches, these disjunctions were generally poorly understood. During the past two decades, however, the phylogenetic relationships between disjunct lineages, the timing of these disjunctions, and putative migration pathways for many disjunct taxa have been elucidated using molecular data and new analytical techniques [4–6]. Most of these studies have focused on woody plants, but several studies have examined the evolution of these disjunct patterns in terrestrial herbs [7–11].

The primary hypothesis put forth for explaining patterns of East Asian/eastern North American floristic disjunctions has been that a once continuous Arcto-Tertiary flora existed in the Northern Hemisphere during the late Cretaceous and Palaeogene that was fragmented by extinction due to global climatic cooling during the Neogene and Quaternary [3,12–14]. However, the wide range of divergence times estimated from molecular dating among disjunct taxa between eastern Asia and North America suggests multiple and complex origins of the disjunctions in the Northern Hemisphere [15]. Based on 98 lineages with disjunct distributions between the two regions, Wen et al. [6] hypothesized that most of these lineages originated in eastern Asia and subsequently moved to North America, but also postulated that some have migrated in the opposite direction. At the same time, several groups present a distinct pattern, such as *Triosteum* L. (Carprifoliaceae), *Viburnum* L. (Adoxaceae), *Astilbe* Buch.-Ham. ex D. Don (Saxifragaceae), and *Meehania* Britt. ex Small et Vaill. (Lamiaceae), with the Tertiary Arctic being the putative center of origin for these taxa [7,16,17]. The Arcto-Tertiary flora once occupied wide areas of northern high latitudes in Cretaceous and early Paleogene time [18,19], and this vegetation subsequently migrated southward to middle latitudes in Eurasia and North America [20]. During such movements in space and time, many taxa became extinct or restricted to central and southern China and/or eastern/western North America. However, the Arcto-Tertiary biogeographic hypothesis alone cannot explain the disjunct distribution of many taxa because of plant migration during more recent times. Two migration routes, the Bering land bridge (BLB) and the North Atlantic land bridge (NALB), are crucial in interpreting Northern Hemisphere floristic disjunctions [21–24]. Paleontological and molecular data suggest that the BLB was used mostly by temperate taxa prior to the late Miocene (<10 Mya) [6,13,15], while the NALB has been viewed as a crucial route for the spread of subtropical and tropical taxa in the early Paleogene [13,23,25]. Recently, the transoceanic long distance dispersal (LDD) has been proposed for taxa for which no land migration route existed at the time of migration, e.g. *Kelloggia* Torrey ex Benth. & J. D. Hooker of Rubiaceae [9] and *Leibnitzia* Cass. of Asteraceae [26].

**Fig 1:**
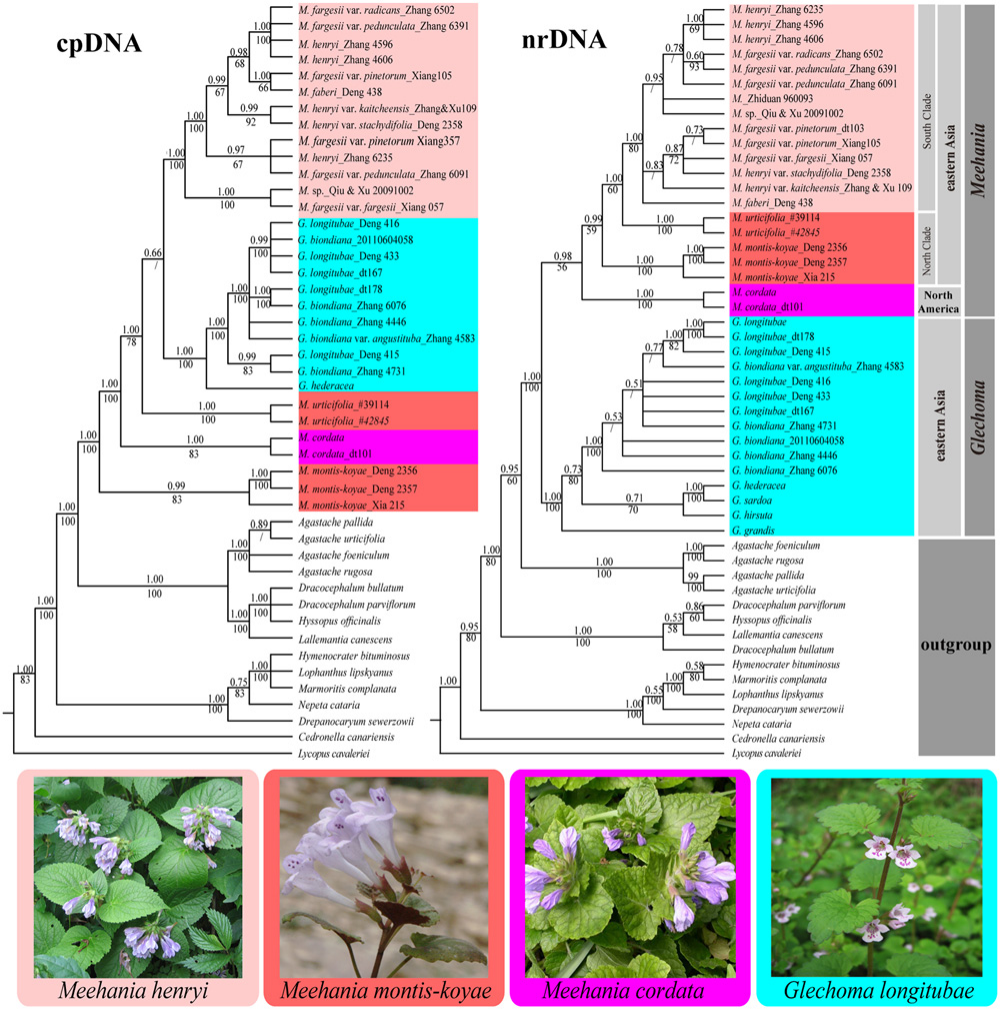
The Bayesian 50% majority-rule consensus tree of *Meehania* and closely related taxa inferred from analyses using (right) combined nuclear ribosomal DNA regions (ITS and ETS) and (left) combined chloroplast DNA regions (*rbcL*, *rps16*, *trnL-F*, *rpl32-trnL* and *psbA-trnH*). Numbers above the nodes are Bayesian posterior probabilities and below the nodes are bootstrap values obtained from MP analysis.

*Meehania* is a small genus of annual and perennial herbaceous plants consisting of seven species and five subspecies [27]. *Meehania* has an unevenly disjunct distribution between eastern Asia (11 taxa) and eastern North America (1 taxon; Fig. 1). Perhaps in part due to its disjunct distribution, *Meehania* species were previously assigned to distant genera such as *Dracocephalum* L., *Cedronella* Moench, and *Glechoma* L. [27]. To date, the taxonomy of the genus, particularly the eastern Asian species, has only been assessed based on morphology. Morphological variation within *Meehania* is chiefly observed in inflorescences, calyx characters, and especially leaf morphology [27–29]. According to our field investigations and specimen examinations, however, leaf morphology is highly variable in different populations.

The genus *Meehania* is characterized by having stolons, cordate-ovate to lanceolate leaves, thyrsoid, terminal cymes, a pedunculate or sessile inflorescence with larger flowers (ca. 1–2.5 cm long), a tubular calyx, a strongly 2-lipped and 5-lobed (3/2) corolla, and parallel anther-thecae [27,31]. Cytological analyses based on two species of *Meehania*, *M*. *urticifolia* (Miq.) Makino and *M*. *montis-koyae* Ohwi, indicated that the genus is diploid, 2n = 18 [30]. *Meehania*, together with 12 other extant genera, belongs to subtribe Nepetinae, tribe Mentheae, but its systematic position within the subtribe is uncertain [31]. Although significant progress has been made in Lamiaceae phylogenetics at the tribal and generic levels [32–38], the genus *Meehania* has been underrepresented in molecular systematic studies. Thus far, only two molecular phylogenetic studies have included *Meehania* species [34,39]. In their study on tribe Mentheae, *Meehania* was included as a member of the subtribe Nepetinae by Drew and Sytsma [34]. They suggested that *Meehania* was polyphyletic because of the inclusion of the Eurasian genus *Glechoma* and Chinese endemic *Heterolamium* C. Y. Wu. However, their sampling was limited as their study only included two species of *Meehania* and one species of *Glechoma*. Furthermore, the voucher specimen for *Heterolamium debile* (Hemsl.) C. Y. Wu (*Zhiduan*, *960093*) used in their study was subsequently found to be misidentified by the first author of this paper, and is in fact *M*. *henryi* (Hemsl.) Sun ex C. Y. Wu. Therefore, a comprehensive species sampling of both *Meehania* and *Glechoma* is vital for resolving relationships within and between the two genera.

Although *Meehania* is not especially species-rich compared with some other well-known Nepetoideae genera (e.g. *Salvia* L., *Nepeta* L.), its East Asian/North American disjunct distribution makes it well suitable for testing the hypothesis that Arctic latitudes in the Tertiary were a major center of origin for taxa currently occurring in East Asia and elsewhere in the North Hemisphere. It is noteworthy that of the ~12 genera of subtribe Nepetinae, 3 possess analogous East Asian/North American disjunct distributions, suggesting common migration routes and similar evolutionary processes in these genera. *Meehania* species typically occur in temperate to subtropical forests in the Northern Hemisphere. In eastern Asia, *M*. *urticifolia* and *M*. *montis-koyae* are both restricted to northeastern China and Japan in temperate areas [27,28,40], while the other four species, *M*. *faberi* (Hemsl.) C. Y. Wu, *M*. *pinfaensis* (H. Lév.) Sun ex C. Y. Wu, *M*. *fargesii* and *M*. *henryi*, are widespread in areas to the south of the Yangtze River in China [27,40]. In these southerly areas, *Meehania* taxa inhabit mesic sheltered microhabitats within coniferous or mixed evergreen broad-leaved forests in moist alpine areas and along valley streams. The perennial *M*. *cordata* (Nutt.) Britt. is endemic to eastern North America, and ranges from Southwest Pennsylvania in the north to North Carolina in the south, and is found as far west as southern Illinois. Few mints exhibiting a primarily East Asian-eastern North American disjunction pattern have been the primary focus of phylogenetic or biogeographic studies. Thus, *Meehania* offers an excellent opportunity to study biogeography and diversification of an East Asian/North American disjunct group distributed across the temperate and subtropical regions of two continents.

In order to test the hypothesis of an Arcto-Tertiary origin of *Meehania* and subsequent migration southward to south-central China and south-eastern North America, we collected accessions of *Meehania* throughout its range and employed DNA sequence data from both the nuclear ribosomal and chloroplast genomic regions to address the following specific questions: (1) Is *Meehania* monophyletic, and how is it related to *Glechoma* and other genera of Nepetinae? (2) When and where did *Meehania* evolve? and (3) what was the likely mechanism or route that facilitated the East Asian/eastern North American disjunction within the genus?

## Materials and Methods

### Ethics Statement

The authors have studied herbarium materials from the herbaria KUN and PE. No special permits were required for this study because all samples were collected by researchers with introduction letters of KIB (Kunming Institute of Botany, Chinese Academy of Sciences) in Kunming. Voucher specimens were deposited in the Herbarium, Kunming Institute of Botany, CAS (KUN). The plant materials did not involve endangered or protected species.

### Taxon sampling

A total of 19 accessions belonging to 11 of the 12 currently recognized taxa of *Meehania* were included in this study (Table 1). Only *M*. *pinfaensis* (Levl.) Sun ex C. Y. Wu, a narrow endemic from Guizhou Province of southwestern China, was not sampled. Our sampling of *Meehania* covered the whole geographic range of the genus from southern and northern East Asia and eastern North America. All samples of *Meehania* in this study were wild collected and dried with silica-gel except for two accessions of *M*. *urticifolia* obtained from herbarium specimens (Table 1). As recent phylogenetic studies of Mentheae show that *Glechoma* is the closest relative to *Meehania* [34,39], 10 accessions of *Glechoma* were included in this study (Table 1). Sequences of two *Meehania* and five *Glechoma* accessions from GenBank were also included in our analyses (S1 Appendix).

**Table 1:** List of species/taxa with voucher information (Herbarium), location, GenBank accession for sequences of species used in this study.

Taxa	Voucher	ITS	ETS	trnL-F	rpl32-trnL	psbA-trnH	rbcL	rps16
*Eriophyton wallichii* Bentham	*SNJ Exped*. *20110814032* (KUN)	KM886719	KM886684	KM886612	KM886814	---	---	---
*Glechoma biondiana* var. *angustituba* C. Y. Wu & C. Chen	*D*. *G*. *Zhang 4583* (KUN)	KM886720	KM886685	KM886613	KM886815	KM886752	KM886782	KM886652
*G*. *longituba* (Nakai) Kuprianova	*dt 178* (KUN)	KM886721	KM886686	KM886614	KM886816	KM886753	KM886783	KM886653
*G*. *longituba* (Nakai) Kuprianova	*T*. *Deng 415* (KUN)	KM886722	KM886687	KM886615	KM886817	KM886754	KM886784	KM886654
*G*. *longituba* (Nakai) Kuprianova	*T*. *Deng 416* (KUN)	KM886723	KM886688	KM886616	KM886818	KM886755	KM886785	KM886655
*G*. *longituba* (Nakai) Kuprianova	*T*. *Deng 433* (KUN)	KM886724	KM886689	KM886617	KM886819	KM886756	KM886786	KM886656
*G*. *longituba* (Nakai) Kuprianova	*dt167* (AJOU)	KM886725	KM886690	KM886618	KM886820	KM886757	KM886787	KM886657
*Glechoma biondiana* (Diels) C. Y. Wu & C. Chen	*D*. *G*. *Zhang 4731* (KUN)	KM886726	KM886691	KM886619	KM886821	KM886758	KM886788	KM886658
*Glechoma biondiana* (Diels) C. Y. Wu & C. Chen	*SNJ Exped*. *20110604058* (KUN)	KM886727	KM886692	KM886620	KM886822	KM886759	KM886789	KM886659
*Glechoma biondiana* (Diels) C. Y. Wu & C. Chen	*D*. *G*. *Zhang 4446* (KUN)	KM886728	KM886693	KM886621	KM886823	KM886760	KM886790	KM886660
*Glechoma biondiana* (Diels) C. Y. Wu & C. Chen	*D*. *G*. *Zhang 6076* (KUN)	KM886729	KM886694	KM886622	KM886824	KM886761	KM886791	KM886661
*Hyptis laniflora* Benth.	*B*. *Drew 41* (WIS)	---	---	KM886623	KM886825	---	---	---
*Isodon dawoensis* (Hand.-Mazz.) H. Hara	*Erskine et al*., *392* (UC)	---	---	KM886624	KM886826	---	---	---
*Lavandula angustifolia* Mill.	*J*. *Walker 2565* (WIS)	---	---	KM886625	KM886827	---	---	---
*Lycopus cavaleriei* H.Lév.	*SNJ Exped*. *20110807071* (KUN)	KM886730	KM886695	KM886626	KM886828	KM886762	KM886792	KM886662
*Marmoritis complanata* (Dunn) A. L. Budantzev	*T*. *Deng 2359* (KUN)	KM886731	KM886696	KM886627	KM886829	KM886763	KM886793	KM886663
*Meehania cordata* (Nutt.) Britton	*dt 101* (KUN)	KM886732	KM886697	KM886628	KM886830	KM886764	KM886794	KM886664
*Meehania faberi* (Hemsl.) C.Y.Wu	*T*. *Deng 438* (KUN)	KM886733	KM886698	KM886629	KM886831	KM886765	KM886795	KM886665
*Meehania fargesii* var. *fargesii* (H. Léveillé) C. Y. Wu	*C*. *L*. *Xiang 057* (KUN)	KM886734	KM886699	KM886630	KM886832	KM886766	KM886796	KM886666
*Meehania fargesii* var. *pedunculata* (Hemsley) C. Y. Wu	*D*. *G*. *Zhang 6091* (KUN)	KM886735	KM886700	KM886631	KM886833	KM886767	KM886797	KM886667
*Meehania fargesii* var. *pedunculata* (Hemsley) C. Y. Wu	*D*. *G*. *Zhang 6391* (KUN)	KM886736	KM886701	KM886632	KM886834	KM886768	KM886798	KM886668
*Meehania fargesii* var. *pinetorum* (Handel-Mazzetti) C. Y. Wu	*C*. *L*.*Xiang 056* (KUN)	KM886737	KM886702	KM886633	KM886835	KM886769	KM886799	KM886669
*Meehania fargesii* var. *pinetorum* (Handel-Mazzetti) C. Y. Wu	*C*. *L*.*Xiang 357* (KUN)	KM886738	KM886703	KM886634	KM886836	KM886770	KM886800	KM886670
*Meehania fargesii* var. *radicans*	*D*. *G*. *Zhang 6502* (KUN)	KM886739	KM886704	KM886635	KM886837	KM886771	KM886801	KM886671
*Meehania henryi* (Hemsl.) Y. Z. Sun ex C. Y. Wu	*D*. *G*. *Zhang 4596* (KUN)	KM886740	KM886705	KM886636	KM886838	---	KM886802	KM886672
*Meehania henryi* (Hemsl.) Y. Z. Sun ex C. Y. Wu	*D*. *G*. *Zhang 6235* (KUN)	---	KM886706	KM886637	KM886839	KM886772	KM886803	KM886673
*Meehania henryi* (Hemsl.) Y. Z. Sun ex C.Y.Wu	*D*. *G*. *Zhang 4606* (KUN)	---	KM886707	KM886638	KM886840	KM886773	KM886804	KM886674
*Meehania henryi* var. *kaitcheensis* (H. Léveillé) C. Y. Wu	*D*. *G*. *Zhang &L*. *Xu 109* (KUN)	KM886741	KM886708	KM886639	KM886841	KM886774	KM886805	KM886675
*Meehania henryi* var. *stachydifolia* (H. Léveillé) C. Y. Wu	*T*. *Deng 2358* (KUN)	KM886742	KM886709	KM886640	---	---	KM886806	KM886676
*Meehania montis-koyae* Ohwi	*G*. *H*. *Xia 215* (KUN)	KM886743	KM886710	KM886641	KM886842	KM886775	KM886807	KM886677
*Meehania montis-koyae* Ohwi	*T*. *Deng 2356* (KUN)	KM886744	---	KM886642	---	KM886776	KM886808	KM886678
*Meehania montis-koyae* Ohwi	*T*. *Deng 2357* (KUN)	KM886745	KM886711	KM886643	KM886843	KM886777	KM886809	KM886679
Meehania sp.	*Qiu & Su 20091002* (KUN)	KM886746	KM886712	KM886644	KM886844	KM886778	KM886810	KM886680
*Meehania urticifolia* (Miq.) Makino	*# 39114* (AJOU)	KM886747	KM886713	KM886645	KM886845	KM886779	KM886811	KM886681
*Meehania urticifolia* (Miq.) Makino	*# 42845* (AJOU)	---	KM886714	---	KM886846	KM886780	KM886812	KM886682
*Melissa axillaris* (Bentham) Bakhuizen f.	*SNJ Exped*. *20110809081* (KUN)	KM886748	KM886715	KM886646	KM886847	---	---	---
*Ocimum basilicum* L.	*J*. *Walker 2557* (WIS)	---	---	KM886647	KM886848	---	---	---
*Plectranthus cremnus* B.J. Conn	*H*. *Forbes s*.*n*. (UC)	---	---	KM886648	KM886849	---	---	---
*Prunella vulgaris* L.	*SNJ Exped*. *20110719005* (KUN)	KM886749	KM886716	KM886649	KM886850	KM886781	KM886813	KM886683
*Salvia maximowicziana* Hemsley	*SNJ Exped*. *20110719092* (KUN)	KM886750	KM886717	KM886650	KM886851	---	---	---
*Salvia scapiformis* Hance	*SNJ Exped*. *20110606022* (KUN)	KM886751	KM886718	KM886651	KM886852	---	---	---

Based on previous phylogenetic studies of the tribe Mentheae [34,39], *Agastache* Clayt., *Cedronella* Moench, *Dracocephalum*, *Drepanocaryum* Pojark., *Hymenocrater* Fisch. & C.A. Mey., *Hyssopus* L., *Lallemantia* Fisch. et Mey., *Lophanthus* Adans., *Marmoritis* Benth., and *Nepeta* L. from subtribe Nepetinae were also included in this study, and *Lycopus* L. was used as an outgroup for our phylogenetic analyses.

In addition to the taxon sampling above, we also sampled across the Nepetoideae for our divergence time analyses (see below). Voucher information and GenBank accession numbers for all specimens used in this study are listed in Table 1, as well as S1 Appendix.

### DNA extractions, amplification, and sequencing

Total genomic DNA was isolated from silica gel-dried leaf material using a Universal Genomic DNA Extraction Kit (Takara, Dalian, China). Five chloroplast (*rbcL*; the *rps16* intron; the *trnL*-*F* region; the *rpl32*-*trnL* and *psbA*-*trnH* intergenic spacers) and two nuclear ribosomal regions (ITS and ETS) were selected for phylogenetic inference. Primers used for amplification and sequencing were Z1 and 1204R for *rbcL* [41], F and 2R for the *rps16* intron [42], and tabc and tabf [43] for the *trnL-F* region. The *rpl32-trnL* and *psbA-trnH* spacers were amplified using the primers as described by Shaw et al. [44] and Sang et al. [45], respectively. ITS was amplified and sequenced using the primers ITS1 and ITS4 [46], and ETS was amplified and sequenced as described in Drew and Sytsma [39]. Amplified DNA samples were analyzed by electrophoresis on 1.4% agarose gel, run in a 0.5 × TBE buffer and detected by ethidium bromide staining. The PCR products were then purified using a QiaQuick gel extraction kit (Qiagen, Inc., Valencia, California, USA) and directly sequenced in both directions using the amplification primers on an the ABI 3730 automated sequencer (Applied Biosystems, Forster City, California, USA).

### Sequence alignment and phylogenetic analyses

DNA Baser v.3 (http://www.DnaBaser.com) was used to evaluate the chromatograms for base confirmation and to edit contiguous sequences. Multiple-sequence alignment was performed by MAFFT v.6 [47], using the default alignment parameters followed by manual adjustment in Se-Al v2.0a11 (http://tree.bio.ed.ac.uk/software/seal/), and gaps were treated as missing data.

Phylogenetic trees were constructed using maximum-parsimony (MP) and Bayesian inference (BI). The MP analyses were conducted using PAUP* version 4.0b10 [48]. All characters were weighted equally and unordered. Most parsimonious trees were searched with a heuristic algorithm comprising tree bisection-reconnection, branch swapping, MULPARS, and the alternative character state. A strict consensus tree was constructed from the most parsimonious trees. Bootstrap analyses (BP; 1000 pseudoreplicates) were conducted to examine the relative level of support for individual clades on the cladograms of each search [49].

Nucleotide substitution model parameters were determined for cpDNA and nrDNA data sets using MrModeltest version 2.3 [50,51]. Bayesian inference was conducted using MrBayes version 3.2.1 [38,52] with the model parameters determined from MrModeltest. For the chloroplast DNA partitions MrModeltest suggested the K81uf+ Γ (*rps16*, *psbA-trnH* and *trnL-F*) and TVM+ Γ (*rbcL* and *rpl32-trnL* spacer) models. For the nrDNA partitions, MrModeltest suggested the TVM+ Γ model for ETS and GTR+ I+ Γ for ITS. The Markov chain Monte Carlo (MCMC) algorithm was run for 3,000,000 generations with one cold and three heated chains, starting from random trees and sampling one out of every 300 generations. Runs were repeated twice to test the convergence of the results. The burn-in and convergence diagnostics were graphically assessed using AWTY [53]. After discarding the trees saved prior to the burn-in point (ca. 15%), the remaining trees were imported into PAUP and a 50% majority-rule consensus tree was produced to obtain posterior probabilities (PP) of the clades. The incongruence length difference (ILD) test [54] was used to evaluate congruence between the chloroplast and the nuclear data sets. For all ILD tests, 100 replications were performed using PAUP*. As the ILD test (P < 0.01) suggested incongruence between the two data sets, and the topologies also exhibited discordance, we performed separate analyses for the cpDNA and the nrDNA data.

### Divergence time estimation

For our divergence time estimation, we analyzed the *Meehania* clade within a broad phylogenetic framework of Lamiaceae to enable multiple fossil calibrations. We included 79 taxa from Nepetoideae in our nrDNA dataset and 74 Nepetoideae taxa for the cpDNA dataset, of which 59 were obtained from GenBank (S1 Appendix). *Eriophyton wallichii* Benth. from the Lamioideae served as an outgroup.

Like most plant groups, the fossil record of Lamiaceae is fairly sparse [31], but there are several described fossils that are useful for calibration points. Hexacolpate pollen is a synapomorphy for subfamily Nepetoideae [31], but is otherwise very rare within angiosperms. Kar [55] identified a middle Eocene hexacolpate pollen sample as *Ocimum* L., which is within the Ocimeae tribe of Nepetoideae. However, based upon the comments of Harley et al. [31], we followed the methodology employed by Drew and Sytsma [34] and placed the fossil calibration at the crown of Nepetoideae as opposed to elsewhere (crown of the Ocimeae). Following the procedure of Drew and Sytsma [34], for both the nrDNA and cpDNA datasets the Nepetoideae crown was constrained with a lognormal prior having an offset of 49 million years (Mya), a mean of 2.6, and a standard deviation (SD) of 0.5. In both datasets we also constrained the most recent common ancestor of *Melissa* L. and *Lepechinia* Willd. with a log-normal distribution having an offset of 28.4 Mya, a mean of 1.5, and a SD of 0.5. The offset was based on a fossil fruit of *Melissa* from the early-middle Oligocene [56,57]. Additionally, *Lepechinia* and *Melissa* were constrained to be monophyletic in both the nrDNA and cpDNA analyses. To prevent the root of the tree from “running away” [58], the root of both the nrDNA and cpDNA trees was constrained using a uniform prior distribution with a minimum of 49 Mya and a maximum of 84 Mya. The maximum age corresponded to the upper age estimate (from the 95% HPD) obtained for the family Lamiaceae in Drew and Sytsma [34]. Since the oldest crown date for the order Lamiales is 107 Mya [34,59], and the Lamiaceae is nested deeply within the Lamiales, the 84 Mya maximum age for Lamiaceae used here is conservative.

Bayesian dating based on a relaxed-clock model [60] was used to estimate the divergence times of the main clades in *Meehania* using the program BEAST version 1.8.0 [61]. BEAST employs a Bayesian MCMC approach to co-estimate topology, substitution rates and node ages [62]. Based on the results from Modeltest, the nrDNA analyses were performed using the GTR model of nucleotide substitution with a Γ and invariant sites distribution with six rate categories, while for the cpDNA data the TVM + Γ model was employed. The tree prior model (Yule) was implemented in the analysis, with rate variation across branches assumed to be uncorrelated and lognormally distributed [60]. Posterior distributions of parameters were approximated using two independent MCMC analyses of 30,000,000 generations (sampling once every 5000 generations). Samples from the two chains, which yielded similar results, were combined after a 10% burn-in for each. Convergence of the chains was checked using the program Tracer 1.5 [63], and the effective sample size (ESS) was well over 200 for all categories.

### Biogeographic analyses

Analysis of potential ancestral distribution areas of clades and taxa in *Meehania* was conducted using RASP 2.1b [64], which implements the S-DIVA (statistical dispersal-vicariance analysis) method [65]. The input file for RASP consisted of the 10,800 post-burn-in trees from our nrDNA BEAST analyses. Three areas of endemism were defined for the biogeographical analysis based on the extant distribution of the genus and the geological history: A, northeastern Asia; B, southeastern Asia; C, eastern North America. Because there were no species in our studied taxa distributed in more than two areas, the maximum range size was constrained to 2 in our analyses.

## Results

### Phylogenetic analyses

The combined nrDNA data matrix had 1144 characters, 519 of which were variable and 339 were potentially parsimony-informative. The parsimony strict consensus tree was largely congruent with the Bayesian consensus tree, especially concerning the backbone of the *Meehania* phylogeny. The Bayesian consensus tree with PP and BP values is shown in Fig. 1 (right). The combined chloroplast DNA (*rbcL*, *rps16*, *trnL-F*, *rpl32-trnL* and *psbA-trnH*) matrix consisted 4727 of characters, of which 914 were variable and 426 potentially parsimony-informative. Topologies from the parsimony strict consensus tree and the Bayesian tree are largely congruent, and the Bayesian tree with PP value and BP support is shown in Fig. 1 (left).

Phylogenetic analysis based on the nrDNA data supported the monophyly of *Meehania* (Fig. 1). In the nrDNA tree, all *Glechoma* taxa formed a clade sister to a clade of *Meehania* species with strong support (Fig. 1, BP = 100, PP = 1.00). By contrast, in the cpDNA tree, *Glechoma* was nested within (instead of sister to) the *Meehania* clade, and was sister to the southeastern Asian *Meehania* clade, but this relationship received weak Bayesian support (PP = 0.67) and no parsimony support (Fig. 1).

Within *Meehania*, four lineages were well recognized in both the nuclear and chloroplast datasets: *M*. *cordata* (North America), *M*. *montis-koyae* (Japan and East China), the *M*. *urticifolia* (Northeast Asia), and a clade including the remaining species from southeastern Asia. The phylogenies resulting from the cpDNA analysis showed that *M*. *montis-koyae* diverged first, whereas in the nuclear data analysis, *M*. *cordata* was the first-diverging lineage. Both nuclear and chloroplast results indicated phylogenetic relationships among *M*. *henryi*, *M*. *fargesii*, and *M*. *faberi* are uncertain.

### Biogeographic analysis

**Fig 2:**
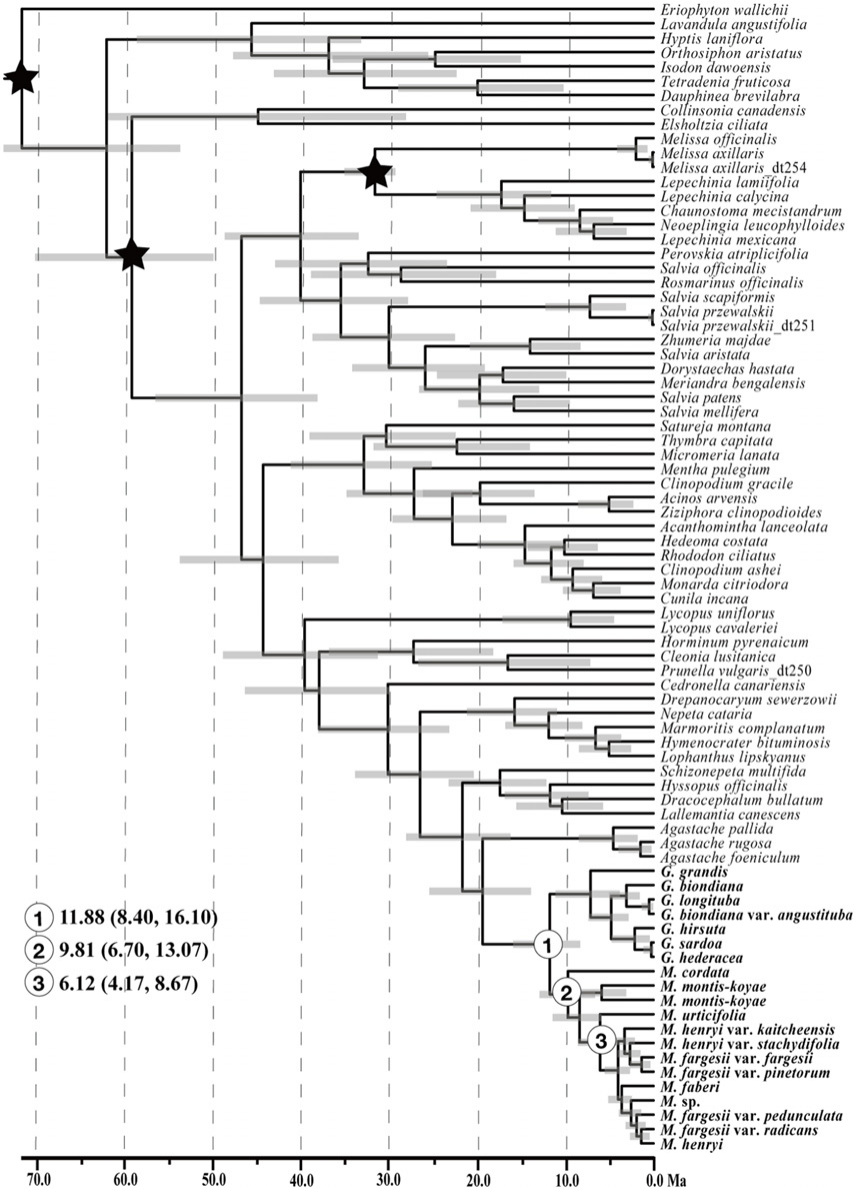
The results of BEAST analysis based on combined nrITS and nrETS data. Gray bars represent the 95% highest posterior density intervals for node ages. Numerals 1–3 are nodes of interests as discussed in the text, and fossil calibrations are marked with black stars.

The chronogram and results of divergence-time estimation based on the nrDNA are shown in Fig. 2. The divergence age between *Meehania* and its sister *Glechoma* was estimated at 11.88 Mya with 95% highest posterior density (HPD) of 8.40–16.10 Mya (node 1, Fig. 2). The crown age of *Meehania* (node 2, Fig. 2), indicating the disjunction of *Meehania* between eastern Asia and North America, was estimated at 9.81 Mya in the Miocene (95% HPD 6.70–13.07 Mya). The split between the southeastern Asian *Meehania* lineage from its northern relatives (node 3, Fig. 2) was estimated at 6.12 Mya (95%HPD: 4.17–8.67 Mya). Divergence time estimates based on the cpDNA generated very similar divergence time as those from nrDNA. The crown age of *Meehania* (including *Glechoma*) was estimated to be 11.7 Mya (95%HPD: 7.69–16.72; S1 Fig.). The disjunction between eastern North American *M*. *cordata* and eastern Asian species was estimated to be 7.58 Mya (95%HPD: 4.90–10.86; S1 Fig.).

**Fig 3:**
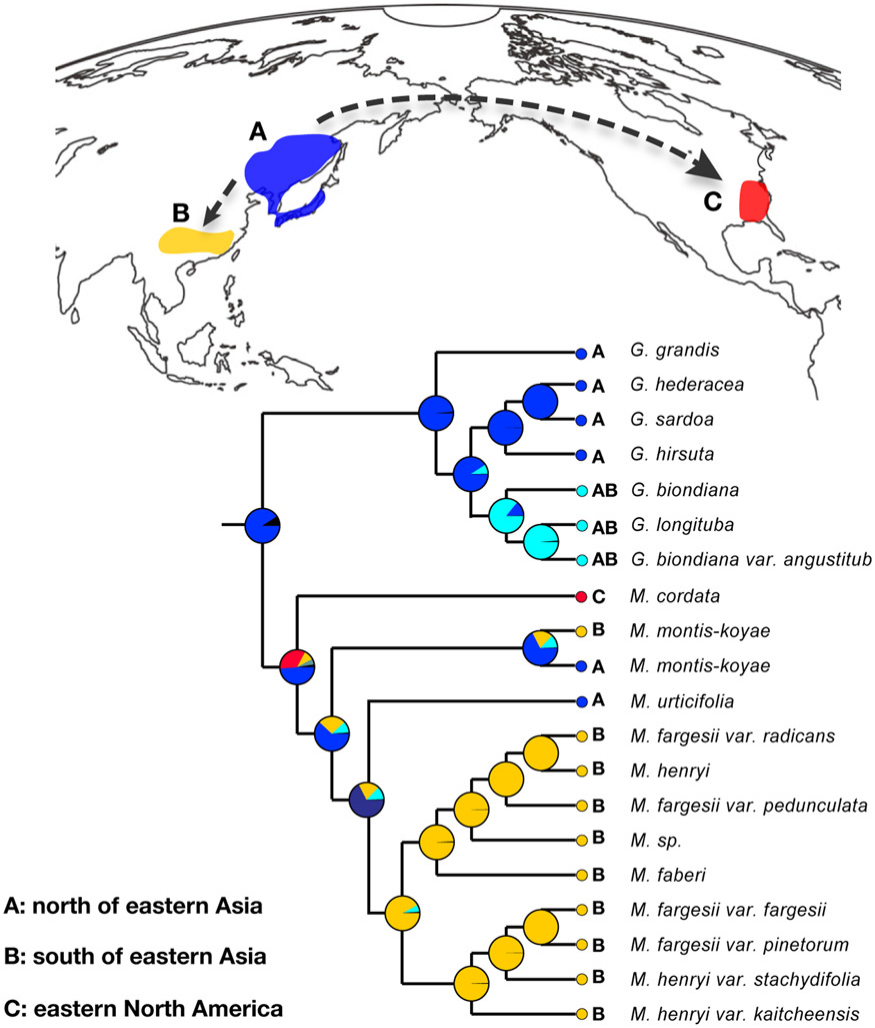
The results of RASP ancestral area reconstruction analysis based on combined nrITS and nrETS data. Three areas of endemism are defined: A (green), north of eastern Asia; B (yellow), south of eastern Asia; C (red), eastern North America. Pie charts show probabilities of ancestral area reconstructions.

In Fig. 3 we illustrate the results obtained from S-DIVA, as well as migration or dispersal routes. The results of the biogeographic inference indicated that the crown node of *Meehania* unequivocally originated in the northern part of eastern Asia. Following the crown divergence, the genus was found to have had two diversification routes: one is an early split from northeastern Asia to eastern North America between *M*. *cordata* and the remaining *Meehania* species; another is a north to south migration within eastern Asia (Fig. 3).

## Discussion

### A reticulate evolutionary history of *Meehania*-*Glechoma* with chloroplast capture

The chloroplast and nuclear phylogenetic analyses produced conflicting results with respect to generic relationships in the subtribe Nepetinae (Fig. 1). The most striking difference between the two topologies is in the position of *Glechoma* and *Meehania cordata*. In the chloroplast DNA tree, species of *Glechoma* formed a well-supported clade embedded within *Meehania* (Fig. 1; BP = 78, PP = 1.0), and sister to the south clade (Fig. 1; PP = 0.66), the pattern found also by Drew and Sytsma [34,39] using chloroplast data and limited sampling of these two genera. In contrast, the nuclear topology clustered all members of *Meehania* as a single moderately-supported clade (Fig. 1; BP = 56, PP = 0.98) and separated the *Glechoma* clade from *Meehania* with high support (Fig. 1; BP = 100, PP = 1.0).

Discordance between nuclear and cytoplasmic data is common in plants [66–69]. One possible explanation for the conflicts has invoked introgression of the cytoplasmic genome from one species into the nuclear background of another (or vice versa) by interspecific hybridization [67,70], in which case the incongruent trees represent the different histories of cp- and nrDNA. Another possible cause is intra-individual polymorphism of nrDNA, which may arise through incomplete concerted evolution, and can cause paralogy problems or incomplete lineage sorting of nrDNA [71].

Morphological data can often be employed in explaining the conflicts between nrDNA and cpDNA topologies [72,73]. The morphological evidence from *Meehania* and *Glechoma* is congruent with their phylogenetic relationships based on the nuclear data. Numerous morphological synapomorphies support *Glechoma* as a separate genus distinguished from *Meehania* in having small flowers (ca. 1–2.5 cm long) in the axils of the middle and upper leaves, an indistinctly 2-lipped calyx, and anther-thecae divaricate at 90° [27, 30]. Since the chloroplast-based phylogeny does not accurately reflect their morphological relationships, the discordance between nrDNA and chloroplast data may be explained by chloroplast capture [66,74]. This inference is common for the mint family [35,72,75], and is specifically shown in such genera as *Phlomis* L. [76], *Sideritis* L. [77], *Bystropogon* L’Hér. [72], *Chelonopsis* Miq. [78], *Conradina* A. Gray [79,80], *Dicerandra* Benth. [81] and *Mentha* L. [82]. Ancient hybridizations with chloroplast introgression may have occurred among ancestors of these isolated taxa.

### Phylogenetic relationships

Based on nrDNA results, two well-supported lineages were recognized within *Meehania*: one clade consists of the single species from eastern North America and the other contains all eastern Asian taxa (Fig. 1). Within the eastern Asian group, the geographically isolated *M*. *montis-koyae* is sister to the remaining species. *Meehania montis-koyae* is endemic to Japan and known only from the type locality in Mt. Koya in Kii Peninsula, Wakayama Prefecture. A suite of morphological characters found in *M*. *montis-koyae*, such as an erect and herbaceous habit, a height of 10–20 cm, abaxial leaves purple, a violet tubular calyx, and an arrangement of flowers in axillary pairs are quite unique within *Meehania*. Recently, Xia and Li [83] reported that *M*. *montis-koyae* is also found in eastern China and occurs on slopes within or at the edge of mixed forests. This plant was previously unknown from China and bridges the two distribution areas between China and Japan. The *M*. *montis-koyae* individual from China is closely related to the two Japanese individuals as inferred by our molecular data with high support (Fig. 1; BP = 100, PP = 1.0). The current disjunction of *M*. *montis-koyae* between eastern China and Japan might be remant populations left over from a previously existing continuous distribution.

Except for *Meehania montis-koyae* and *M*. *urticifolia*, all the species from southeastern Asia form a well-supported south clade (Fig. 1; BP = 80, PP = 1.0). Phylogenetic relationships of the three species complexes among the south clade remained unresolved (Fig. 1), possibly due to the recent evolutionary radiation of this group. However, taxa from this clade exhibit a wide range of morphological and ecological variations. *Meehania faberi* is a distinct species based on its annual life history, morphological traits such as ovate and fleshy leaves and short inflorescences, and a geographically isolated distribution [27]. The two geographically widespread species complexes, *Meehania henryi* and *M*. *fargesii*, were found to be polyphyletic (Fig. 1). The *Meehania henryi* complex is endemic to a small area of Central China and is characterized by an erect habit, a height of ca. 30–60 cm, large leaves, a narrowly tubular calyx, and verticillasters in terminal and lateral racemes [27,40]. The *Meehania fargesii* complex is characterized by having slender stems, a prostrate or stoloniferous habit, a height of 10–20 cm, a tubular calyx, and 2-flowered verticillasters inserted in the leaf axils of the upper 2 or 3 leaf pairs of the stem [27,40]. Subtle differences in verticillaster flower number, stem branching pattern and leaf shape were used previously to delimit subspecies within the complex [27]. Ecologically, the *M*. *henryi* complex is distributed in evergreen broad-leaved and mixed forests from 300–700 m in elevation, whereas the *M*. *fargesii* complex is distributed from temperate mixed forests to coniferous forests at a higher elevation from 700 to 3500 m.

### Historical biogeography and divergence times

*Glechoma*, the sister group of *Meehania*, occurs in north temperate areas in Eurasia, and the basal lineages of *Meehania* (*M*. *montis-koyae* and *M*. *urticifolia*) are also largely restricted to northeastern Asia (i.e., Japan, East China, and South Korea) [27,28], making the high latitude area of Eurasia a plausible ancestral area for *Meehania* (Fig. 3). Ancestral area reconstruction with RASP based on our nrDNA phylogeny supported this view, suggesting a *Meehania* origin in the high latitude area of Eurasia, especially northeastern Asia (Fig. 3). This evidence agrees well with the Arcto-Tertiary origin hypotheses, which has been extensively documented [18,84,85]. Subsequently, the decrease of annual mean temperature at northern latitudes provided opportunities for biota dispersal and subdivision [86]. The present distribution of *Meehania* in eastern North America and northeastern and southeastern Asia could result from vicariance of south-migrating populations during climatic oscillation and further fragmentation and dispersal of these populations. This inference is robustly supported by our molecular phylogenetic results, viz. a sister relationship between North American *M*. *cordata* and the clade of East Asian *Meehania* (the latter comprising the two subclades within this area; Fig. 1). Similar cases are found in *Astilbe* Buch.-Ham. ex D. Don, *Cedrus* Trew, *Maianthemum* Web. and *Triosteum* L. in which the southeastern Asian species were found to have their origin in Arcto-Tertiary geofloras [7,17,87,88]. Zhu et al. [7] suggested *Astilbe* had its origin in Japan and subsequently migrated independently to eastern North America, continental Asia, and even to southeastern Asian islands. Based on fossils and molecular data, Qiao et al. [88] suggested an origin of *Cedrus* in high latitudes of Eurasia, and its present distribution in the Mediterranean and Himalayas could result from vicariance of a southward migration during climatic oscillations in the Tertiary.

The estimated divergence times between the *Meehania* lineages from isolated regions completely overlap the timing of Miocene cooling and drying. In the Miocene, a significant global cooling transition occurred at approximately 15–10 Mya [89–91]. This cooling event was proposed to cause southward invasions and displacements of organisms [92]. As a result, four *Meehania* species occur today in the southernmost areas of eastern Asia (Fig. 3). We estimated the divergence of the southern clade (between the northern *M*. *urticifolia* and other southern Asian taxa) at 4.17–8.67 Mya in the late Miocene. Another Miocene climate change emphasized by Savage [92] caused enhanced aridity at middle latitudes of the Northern Hemisphere. In the interior of Eurasia, a drying event occurred at about 8–7 Mya [93,94] that may have caused isolation between *Meehania* in northern and southern East Asia. Extant *M*. *urticifolia* and *M*. *montis-koyae* show preferences to cool and moist habitats [27,95], and are probably relicts that previously inhabited northern regions. This distribution pattern has also been reported for other taxa, such as *Parthenocissus* Planch. [96], *Mitchella* L. [8] and *Astilbe* Buch.-Ham. ex D. Don [7].

The ancestor of eastern North American *Meehania* might have reached North America in the late Miocene, which is supported by our estimation of ca. 9.81 Mya for the divergence between the North American *M*. *cordata* and the East Asian clade (Fig. 2). The North Atlantic land bridge, which largely contributed to the dispersal of more tropical elements, ceased to exist in the middle Miocene [13], and was apparently less suitable for *Meehania* interchange. We favor a hypothesis based on a migration scenario across the Bering land bridge in the late Miocene. North America and Asia were repeatedly connected via the Bering Bridge, with biotic interchange moderated mainly by climatic factors [97]. The Bering land bridge supported exchanges of temperate floras [3], but was ultimately disrupted by a sharp decrease in average temperatures from the Oligocene to the present [91]. In the late Miocene and Pliocene, the colder climate restricted Beringian interchange to mostly cold-adapted species. Decreasing temperatures could have prohibited subsequent interchange of warm adapted taxa, including *Meehania*, between eastern Asia and eastern North America.

*Meehania*, like other taxa from tribe Mentheae, possess mericarps for dispersal. The dispersal ability of these nutlets is usually limited (reviewed by [56,98]), and long distance dispersal between Asia and North America in *Meehania* is highly unlikely. Consequently, as a result of geographic and ecological isolation, diverged *Meehania* lineages likely formed within each aforementioned isolated region after these climatic change events. These results suggest that vicariance played an important role in the evolution of herbaceous plants between eastern Asia and North America.

## Conclusions

Two important conclusions stem from this study. First, we show that Arctic latitudes were a major center of origin for taxa currently occurring in East Asia and elsewhere in the North Hemisphere. Secondly, the current disjunct distribution of some herbs with a putative Arcto-Tertiary origin is probably a result of vicariance and subsequent southward migration of populations during climatic oscillations in the middle Miocene with subsequent migration into eastern North America via the Bering land bridge in the late Miocene.

## Supporting Information

S1 Fig BEAST chronogram based on *trnL-F* and *trnL*-*rpl32* data. Gray bars represent the 95% highest posterior density intervals for node ages.
(TIF)

S1 Appendix List of taxa with accession numbers obtained from GenBank. (DOC)
