# Supplementary figures and images for: Does the Arcto-Tertiary Biogeographic Hypothesis Explain the Disjunct Distribution of Northern Hemisphere Herbaceous Plants? The Case of Meehania (Lamiaceae)
Source: PLoS One. 2015 Feb 6;10(2):e0117171. doi: 10.1371/journal.pone.0117171 (PMC4319762; doi:10.1371/journal.pone.0117171)

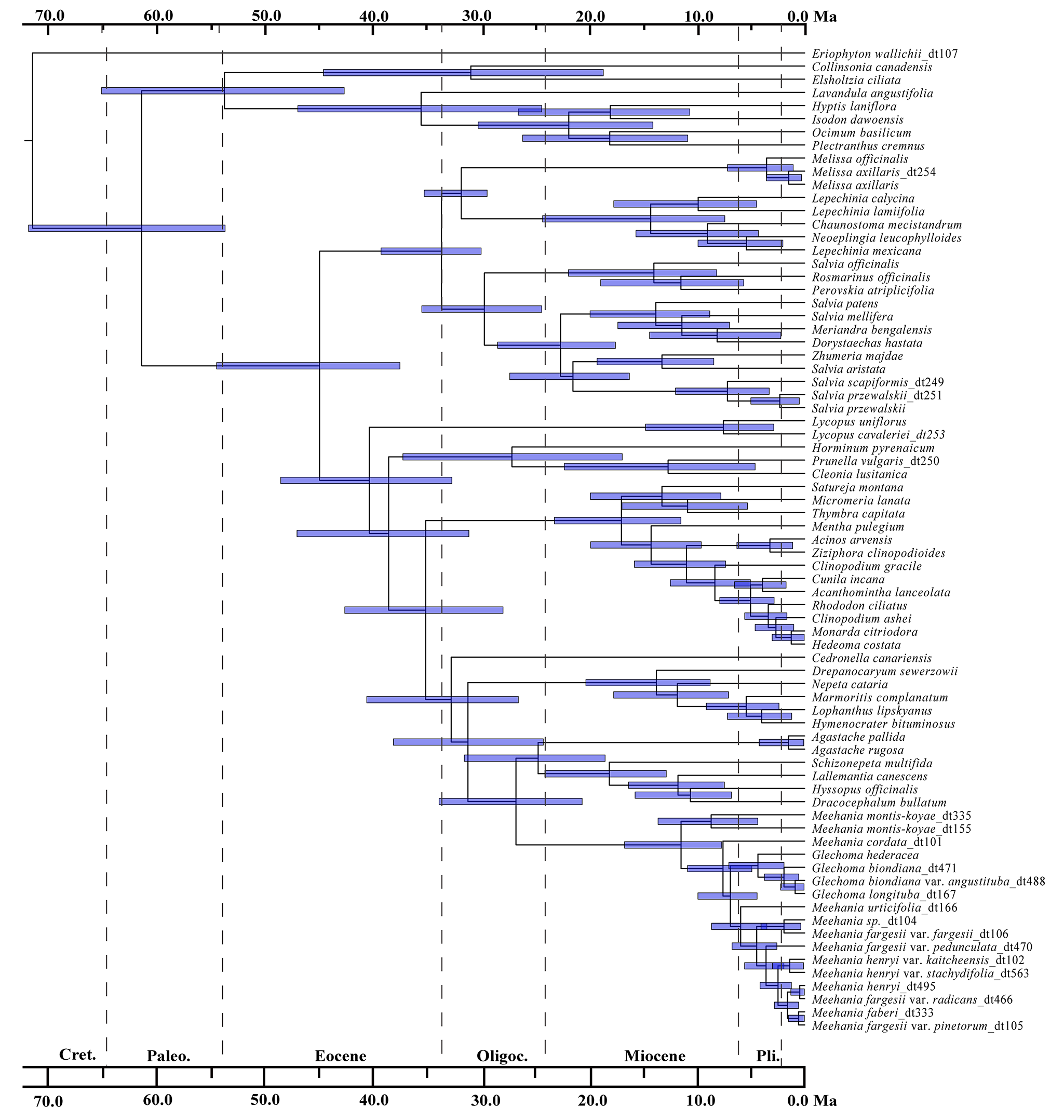

Supplement: S1 Fig — Gray bars represent the 95% highest posterior density intervals for node ages. (TIF) [file pone.0117171.s001.tif]
