# Supplementary material for: Does the Arcto-Tertiary Biogeographic Hypothesis Explain the Disjunct Distribution of Northern Hemisphere Herbaceous Plants? The Case of Meehania (Lamiaceae)
Source: PLoS One. 2015 Feb 6;10(2):e0117171. doi: 10.1371/journal.pone.0117171 (PMC4319762; doi:10.1371/journal.pone.0117171)
Supplement: S1 Appendix — (DOC) [file pone.0117171.s002.doc]

**SUPPLEMENTARY DATA**

**Appendix S1** List of taxa with accession numbers obtained from GenBank.

| **Taxon** | **ITS** | **ETS** | ***trnL-trnF*** | ***rpl32-trnL*** | ***psbA-trnH*** | ***rbcL*** |
| --- | --- | --- | --- | --- | --- | --- |
| *Acanthomintha lanceolata* | DQ667333 | JQ669142 | DQ667522 | JQ669272 | -- | -- |
| *Acinos arvensis* | JQ669074 | JQ669143 | JQ669021 | JQ669273 | -- | -- |
| *Agastache foeniculum* | AY506660 | -- | AY506626 | -- | -- | Z37381 |
| *Agastache pallida* | JQ669075 | JQ669144 | JF301357 | JQ669274 | -- | -- |
| *Agastache rugosa* | JQ669076 | JQ669145 | JQ669022 | JQ669275 | EU590857 | HM590067 |
| *Agastache urticifolia* | DQ667247 | -- | AY570452 | -- | DQ667357 | -- |
| *Cedronella canariensis* | JQ669079 | JQ669148 | JF301360 | JQ669281 | -- | HM849871 |
| *Chaunostoma mecistandrum* | JF301342 | JF301311 | JF301361 | JQ669282 | -- | -- |
| *Cleonia lusitanica* | DQ667309 | -- | DQ667495 | -- | -- | -- |
| *Clinopodium ashei* | DQ667237 | JQ669150 | DQ667437 | JQ669284 | -- | -- |
| *Clinopodium gracile* | JQ669082 | JQ669152 | JQ669027 | JQ669286 | -- | -- |
| *Collinsonia canadensis* | JQ669087 | JQ669157 | JF301364 | JQ669291 | -- | -- |
| *Cunila incana* | DQ667316 | JQ669160 | DQ667504 | JQ669295 | -- | -- |
| *Dauphinea brevilabra* | JF301403 | JF304193 | -- | -- | -- | -- |
| *Dorystaechas hastata* | DQ667252 | JF301312 | AY570454 | JQ669302 | -- | -- |
| *Dracocephalum bullatum* | JQ669096 | JQ669167 | JF301366 | JQ669303 | -- | -- |
| *Dracocephalum parviflorum* | JQ669097 | JQ669168 | JQ669038 | JQ669304 | -- | -- |
| *Drepanocaryum sewerzowii* | DQ667328 | JQ669169 | DQ667517 | JQ669305 | DQ667413 | -- |
| *Elsholtzia ciliata* | JQ669098 | JQ669170 | JF301367 | JQ669306 | -- | -- |
| *Glechoma longituba* | GQ456147 | -- | -- | -- | JF708216 | -- |
| *Glechoma grandis* | GQ456141 | -- | -- | -- | -- | -- |
| *Glechoma hederacea* | JQ669099 | JQ669171 | JF301368 | JQ669307 | DQ006196 | JN890905 |
| *Glechoma hirsuta* | GQ456148 | -- | -- | -- | -- | -- |
| *Glechoma sardoa* | GQ456149 | -- | -- | -- | -- | -- |
| *Hedeoma costata* | DQ667236 | JQ669174 | JQ669041 | JQ669311 | -- | -- |
| *Horminum pyrenaicum* | DQ667257 | JF301314 | AY570456 | JQ669315 | -- | AY570385 |
| *Hymenocrater bituminosis* | JQ669105 | JQ669179 | JQ669045 | JQ669316 | -- | -- |
| *Hyptis laniflora* | -- | -- | JF301370 | JQ669317 | -- | -- |
| *Hyssopus officinalis* | JQ669106 | JQ669180 | JF301371 | JQ669318 | -- | Z37395 |
| *Isodon dawoensis* | -- | -- | JF301372 | JQ669319 | -- | -- |
| *Lallemantia canescens* | JQ669108 | JQ669182 | JF301373 | JQ669321 | -- | -- |
| *Lavandula angustifolia* | -- | -- | AY570457 | JQ669323 | -- | -- |
| *Lepechinia calycina* | JF301344 | JF301315 | JF301375 | JQ669324 | -- | -- |
| *Lepechinia lamiifolia* | JF301348 | JF301320 | JF301379 | JQ669325 | -- | -- |
| *Lepechinia mexicana* | JF301350 | JF301321 | JF301381 | JQ669326 | -- | -- |
| *Lophanthus lipskyanus* | JQ669109 | JQ669183 | JF301384 | JQ669328 | -- | -- |
| *Lycopus uniflorus* | DQ667302 | JQ669185 | DQ667488 | JQ669330 | -- | -- |
| *Marmoritis complanatum* | JQ669111 | JQ669186 | JQ669049 | JQ669331 | -- | -- |
| *Meehania cordata* | JQ669112 | JQ669187 | JQ669050 | JQ669332 | -- | -- |
| *Melissa axillaris* | JQ669114 | JQ669189 | JQ669051 | JQ669334 | -- | -- |
| *Melissa officinalis* | JF301325 | JQ669335 | JF301386 | JF301353 | -- | -- |
| *Mentha pulegium* | JQ669117 | JQ669192 | JQ669053 | JQ669338 | -- | -- |
| *Meriandra bengalensis* | DQ667329 | JF301326 | DQ667518 | -- | -- | -- |
| *Micromeria lanata* | JQ669120 | JQ669196 | JQ669057 | JQ669342 | -- | -- |
| *Monarda citriodora* | JQ669124 | JQ669200 | JF301388 | JQ669346 | -- | -- |
| *Neoeplingia leucophylloides* | JF301354 | JF301327 | JF301390 | JQ669348 | -- | -- |
| *Nepeta cataria* | JQ669126 | JQ669202 | JF301391 | JQ669349 | DQ667388 | Z37421 |
| *Ocimum basilicum* | -- | -- | AY570462 | JQ669350 | -- | -- |
| *Orthosiphon aristatus* | JF301407 | JF304197 | -- | -- | -- | -- |
| *Perovskia atriplicifolia* | DQ667223 | JF301328 | AY570464 | JQ669352 | -- | -- |
| *Plectranthus cremnus* | -- | -- | JF301393 | JQ669354 | -- | -- |
| *Rhododon ciliatus* | JQ669134 | JQ669210 | JF301397 | JQ669363 | -- | -- |
| *Rosmarinus officinalis* | DQ667241 | JF301329 | AY570465 | JQ669364 | -- | -- |
| *Salvia aristata* | DQ667280 | JF301336 | DQ667465 | JQ669365 | -- | -- |
| *Salvia mellifera* | DQ667220 | JF301338 | DQ667427 | JQ669368 | -- | -- |
| *Salvia officinalis* | JF301355 | JF301332 | JF301398 | JQ669369 | -- | -- |
| *Salvia patens* | DQ667253 | JF301333 | DQ667442 | JQ669370 | -- | -- |
| *Salvia przewalskii* | DQ667254 | JF301339 | DQ667443 | JQ669372 | -- | -- |
| *Satureja montana* | JQ669135 | JQ669211 | JQ669067 | JQ669374 | -- | -- |
| *Schizonepeta multifida* | DQ667313 | JQ669213 | JF301400 | JQ669376 | -- | -- |
| *Tetradenia fruticosa* | JF301406 | JF304201 | -- | -- | -- | -- |
| *Thymbra capitata* | JQ669137 | JQ669214 | JF301401 | JQ669377 | -- | -- |
| *Zhumeria majdae* | DQ667335 | JF301341 | DQ667524 | JQ669381 | -- | -- |
| *Ziziphora clinopodioides* | JQ669140 | JQ669218 | JF301402 | JQ669382 | -- | -- |
